# Real-Time In Vivo Imaging of the Developing Pupal Wing Tissues in the Pale Grass Blue Butterfly *Zizeeria maha*: Establishing the Lycaenid System for Multiscale Bioimaging

**DOI:** 10.3390/jimaging5040042

**Published:** 2019-03-28

**Authors:** Kanako Hirata, Joji M. Otaki

**Affiliations:** The BCPH Unit of Molecular Physiology, Department of Chemistry, Biology and Marine Science, Faculty of Science, University of the Ryukyus, Nishihara, Okinawa 903-0213, Japan

**Keywords:** butterfly wing, color pattern formation, forewing-lift method, lycaenid butterfly, pupal wing epithelium, real-time in vivo imaging, scale formation, whole-wing imaging, *Zizeeria maha*

## Abstract

To systematically analyze biological changes with spatiotemporal dynamics, it is important to establish a system that is amenable for real-time in vivo imaging at various size levels. Herein, we focused on the developing pupal wing tissues in the pale grass blue butterfly, *Zizeeria maha*, as a system of choice for a systematic multiscale approach in vivo in real time. We showed that the entire pupal wing could be monitored throughout development using a high-resolution bright-field time-lapse imaging system under the forewing-lift configuration; we recorded detailed dynamics of the dorsal and ventral epithelia that behaved independently for peripheral adjustment. We also monitored changes in the dorsal hindwing at the compartmental level and directly observed evaginating scale buds. We also employed a confocal laser microscopy system with multiple fluorescent dyes for three-dimensional observations at the tissue and cellular levels. We discovered extensive cellular clusters that may be functionally important as a unit of cellular communication and differentiation. We also identified epithelial discal and marginal dents that may function during development. Together, this lycaenid forewing system established a foundation to study the differentiation process of epithelial cells and can be used to study biophysically challenging mechanisms such as the determination of color patterns and scale nanoarchitecture at the multiscale levels.

## 1. Introduction

The recent advancement of bioimaging methods, including inventions of fluorescent dyes for live-cell staining and those of real-time microscopy technologies, has revolutionized many disciplines of biological and medical sciences. Developmental biology is not an exception because visualization has always been an important issue in developmental biology; it studies ever changing systems that cannot be fully understood by fixed tissue sections. In the seminal transplantation experiments that have proposed important concepts such as organizer, morphogen and induction, Spemann and Mangold (1924) [1] used embryos of two different amphibian species with different coloration to differentiate their fates. Similarly, to trace cell fates in early amphibian embryogenesis, the use of vital dyes was invented in the early twentieth century [2] and has been replaced with fluorescent dyes in the late twentieth century together with the use of confocal laser microscopy [3,4,5]. The development of green fluorescent protein (GFP) and transgenic technologies together further opened a new stage of visualization of developmental systems [6,7,8]. 

In the holometabolous insects such as Diptera (flies and mosquitoes), Coleoptera (beetles) and Lepidoptera (butterflies and moths), which have distinct larval, pupal and adult stages, cells that will construct adult tissues and organs in the future comprise imaginal discs at the early larval stage [9,10]. Imaginal discs are sac-like evaginations of the epidermis. In *Drosophila*, the wing imaginal discs and leg imaginal discs have been studied intensively for their fate specification and expressed genes [9,10]. 

Butterfly wings have some interesting features that other insects, including the fruit fly, do not have at all; two of them are the highly diverse wing color patterns and intricate scale nanoarchitecture for structural colors and they are probably nature’s most extravagant fine arts. In butterflies and moths, the wing imaginal discs expand upon pupation to form the pupal wings that constitute the dorsal and ventral epithelia [11]. The pupal wings are located on the surface of the pupal body, which makes the differentiating wings accessible for researchers. Importantly, butterfly wing color pattern determination and scale nanoarchitecture formation occur in the pupal wing epithelial tissue [11]. Equally important, the butterfly pupal wings are large enough for researchers to manipulate physiologically and image them using a real-time imaging system. 

To make the pupal wing tissues experimentally accessible, we have invented the forewing-lift method [12] and have used it for pharmacological treatments [13], molecular biological analyses [14], physical distortion treatments [15] and real-time imaging of color pattern formation in vivo [16,17,18,19,20]. This method has also been employed for electroporation-mediated transgenesis into pupal wings [21,22]. Using the blue pansy butterfly *Junonia orithya* and a confocal imaging system, we have demonstrated that epithelial cells are elongated vertically and that they are connected with other neighboring cells using horizontal processes, which may be cytonemes [17,19,23]. Moreover, we have recorded slow low-frequency contractions of the entire tissues [16,17,20] and spontaneous long-range low-frequency calcium waves [20], although their functional significance has not been fully elucidated. 

The blue pansy butterfly may be considered one of the nymphalid butterfly models; for example, it is relatively easy to rear from eggs that were obtained from field-caught females and it can tolerate surgical manipulations for physiological experiments, including imaging. However, no biological system is perfect. Although easy to manipulate, the pupal wing of the blue pansy butterfly is much larger than the visual field under a microscope; thus, it is not easy to monitor the whole wing and locate what a researcher is looking for in a wing. Moreover, a nymphalid system does not necessarily represent other families of butterflies such as the family Lycaenidae, which is as diverse as the family Nymphalidae. In fact, lycaenid butterflies have rarely been studied in butterfly developmental biology except some studies from our research group [24,25]. There is a historical trend that color pattern studies have favored nymphalid butterflies since the general scheme for butterfly wing color patterns was established as the nymphalid ground plan [11,26,27]. In addition, nymphalid butterflies such as *Junonia* and *Bicyclus* have concentric eyespots, which are often a target of developmental studies.

To circumvent these problems, in the present study we focused on the pupal wing tissues of the pale grass blue butterfly, *Zizeeria maha*, a small lycaenid butterfly abundantly available throughout a year in Okinawa, Japan. Its rearing method has been established [28], and it has been used for mutagenesis studies [24,25], evolutionary biology [29,30] and environmental science to monitor the biological effects of nuclear pollution [31,32,33,34]. The pupal wings of the pale grass blue butterfly are so small that the whole wing area can be monitored under a conventional microscope. The pupal wings of this butterfly are also amenable for real-time in vivo observations at smaller levels (i.e., compartmental, cellular and subcellular levels), making it possible to integrate information on various size levels. We have already obtained the whole wing developmental images of this species at relatively low resolution [20] and we have shown a snapshot of confocal images of cells [17]. However, more detailed imaging is required to understand the developing wings of this butterfly. Here, we present fundamental real-time in vivo information on the developing pupal wing tissue of this butterfly using a high-resolution bright-field microscope and a confocal laser microscope. Together, we show that this butterfly system is amenable for real-time in vivo imaging to monitor spatiotemporal dynamics at multiscale levels. 

## 2. Materials and Methods

### 2.1. Butterflies

Throughout this study, the pale grass blue butterfly, *Zizeeria maha*, was used. The wild females were caught in Ginowan and Itoman, Okinawa-jima Island, Japan. The offspring individuals from these females (confined in a tank with males from the same collection site) were used. Additionally, offspring individuals from different collection sites were crossed in the laboratory and their next-generation individuals were also used. This species of butterfly is probably one of the most populous butterflies in Okinawa and mainland Japan. This butterfly is monophagous; its larvae eat the creeping woodsorrel, *Oxalis corniculata*. This host plant was also wild harvested. No permission was required to collect and study this species of butterfly and its host plant species in Japan. Experiments were ethically performed according to the Animal Experiment Regulations of the University of the Ryukyus.

**Figure 1:**
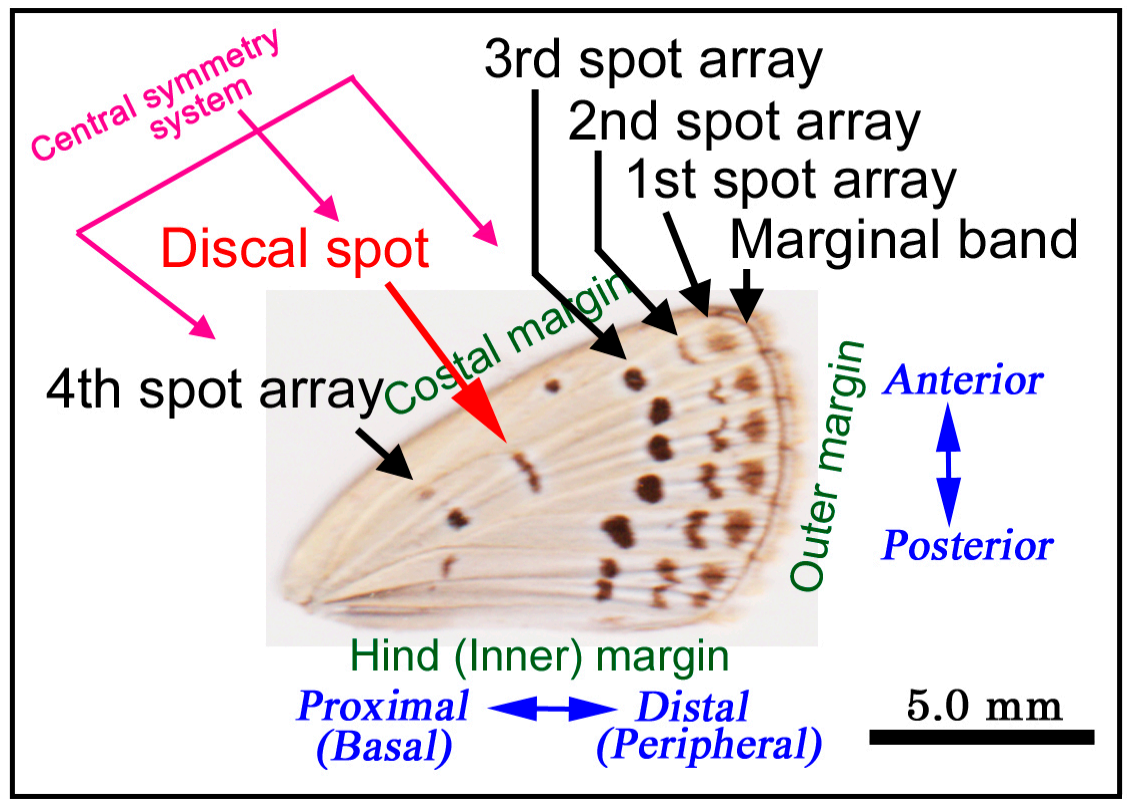
Nomenclature of color pattern elements and other features of the adult forewing of *Z. maha*. The black spot arrays are defined from the distal to proximal direction as the first, second, third and fourth spot arrays. Additionally, there are the marginal band and discal spot. The discal spot is thought to be the center of the central symmetry system that includes the third and fourth spot arrays [24,25].

The nomenclature of the adult forewing spots and other features are shown in Figure 1. The third and fourth spot arrays constitute the central symmetry system around the discal spot [24,25]. It has been speculated based on temperature-shock-induced modifications and mutagenesis that the larval or pupal wing cells located at the prospective discal spot function as the color pattern organizer for the central symmetry system [24,25]. 

### 2.2. Rearing

**Figure A1:**
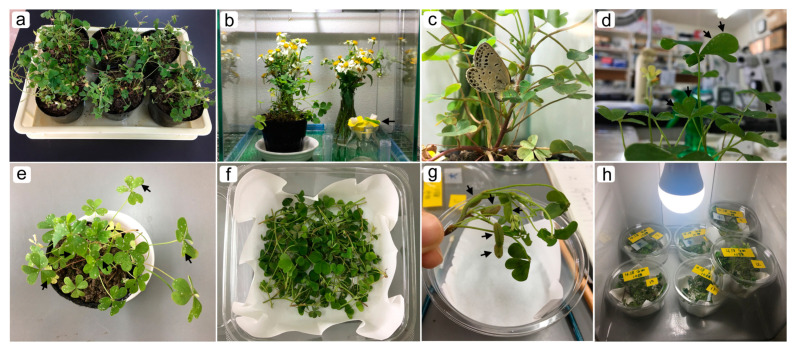
Rearing procedures for *Z. maha*. (**a**) Potted host plant. (**b**) A set up for egg collection from wild-caught females inside a glass tank. (**c**) Oviposition of a female. (**d**) Eggs deposited on the surface of the host plant leaves (arrows). (**e**) Signature of eaten leaves (arrows). (**f**) Host plant leaves inside a plastic container for larval rearing. (**g**) Small plastic container and larvae on the host plant. (**h**) Larval containers inside an incubator set at 13 °C.

We performed the rearing method for the pale grass blue butterfly that has been established [28], with modifications as necessary in this study (Figure A1). Rearing was performed at ambient temperature (26–28 °C) and at ambient humidity (40–80%). Briefly, the host plant was collected in the field and potted individually. The potted host plants together with wild flowers were set within a glass tank Kotobuki Kogei Crystal Cube 300 (W300 mm × D300 mm × H300 mm) (Tenri, Nara, Japan) (Figure A1a). Additionally, an “ion supply drink” POCARI SWEAT (Otsuka Pharmaceutical, Tokyo, Japan) was supplied as artificial nectar for ad-lib access. Field-caught females and males were confined in this tank under strong lighting using metal halide lamps at ambient temperature (26–28 °C) (Figure A1b). After eggs were deposited on the leaves in a few days (Figure A1c,d), the whole plant was moved to a smaller glass tank Kotobuki Kogei Crystal Cube 200 (W200 mm × D200 mm × H200 mm) and kept there for a week or so until the leaf signature eaten by the hatched larvae emerged (Figure A1e). Next, the leaves with larvae were cut at the stalk and transferred to a plastic container (W150 mm × D150 mm × H55 mm) where new leaves were abundantly supplied (Figure A1f). Essentially, larvae were allowed to become prepupae and then pupae at ambient temperature (26–28 °C) and at ambient humidity (40–80%). If necessary, prepupae were put in an incubator set at 4–8 °C for a few hours to a few days to delay pupation due to the availability of microscopes. Pupation was initiated when they were returned to ambient temperature. If necessary, when larvae became the third or fourth instar (Figure A1g), they were reared in a smaller container and put in an incubator at 13 °C to delay their growth and development (Figure A1h). This 13 °C treatment made the developmental time longer (see Results 3.1.); thus, it was indicated when performed.

### 2.3. Surgical Manipulations, Staining and Mounting

**Figure 2:**
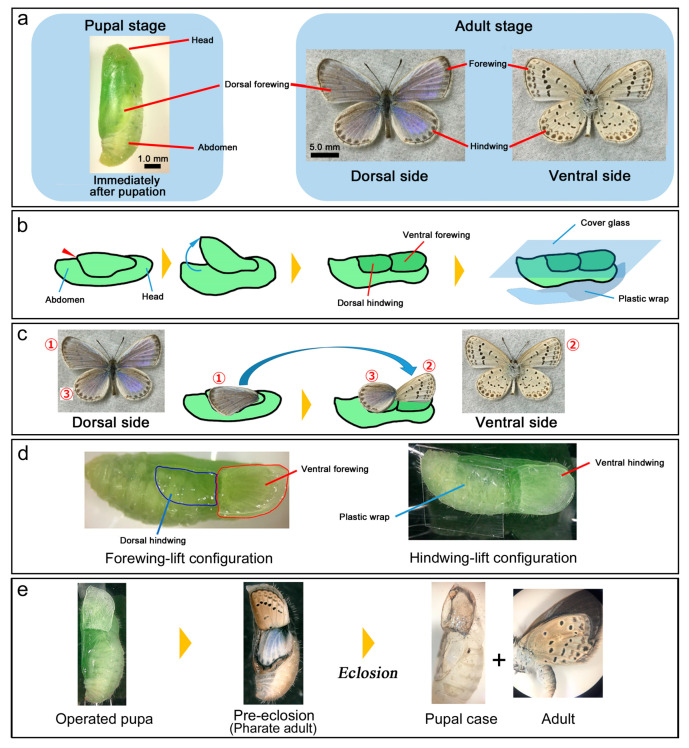
Surgical procedures used in this study. Also see Figure A2; Video S1. (**a**) Pupal and adult morphology. Wings are located on the surface of the pupal body. Hindwings are not seen because they are placed underneath the forewings. Immediately before eclosion, adult forewing color patterns can be seen through the pupal cuticle. (**b**) Forewing-lift operation. The margin of the dorsal forewing is lifted slowly until the ventral forewing and dorsal hindwing can make a single plane. A piece of cover glass is then placed onto these wings. The pupal body is covered and fixed with a piece of plastic wrap from underneath the body. (**c**) Correspondence between the operated pupal wings and adult wings before and after the forewing-lift configuration indicated by numbers. (**d**) A pupa of the forewing-lift configuration (left) and a pupa of the hindwing-lift configuration (right). (**e**) Rough time course of the operated pupa. It develops to the adult stage and often ecloses successfully.

**Figure A2:**
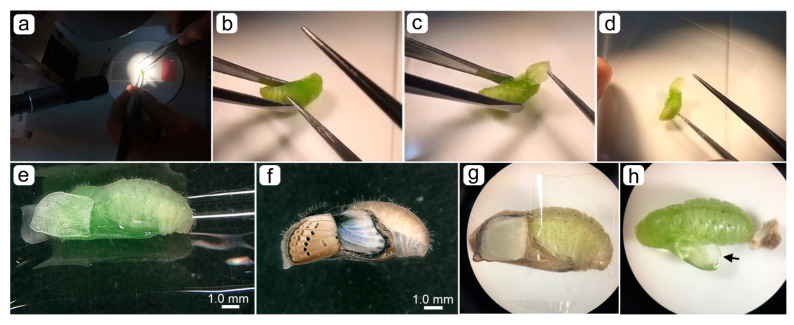
Surgical procedures. See also Figure 2; Video S1. (**a**) Overall view of surgical operation. (**b**) First step: the forewing margin is picked up. (**c**) Second step: the forewing is lifted from the margin to the base. (**d**) Third step: a piece of cover glass is placed on the lifted wings. (**e**) An operated pupa immediately after the surgery in the forewing-lift configuration. (**f**) An operated pupa immediately before eclosion in the forewing-lift configuration. (**g**) An operated pupa in the hindwing-lift configuration. (**h**) An operated pupa for dye loading in the sandwich configuration.

**Figure A3:**
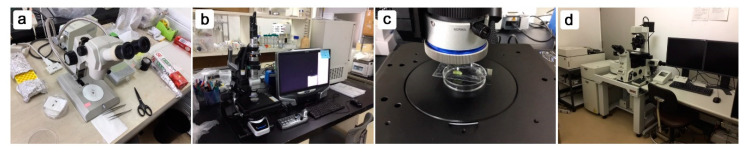
Microscope systems used in this study. (**a**) Conventional dissection stereomicroscope for surgical operations. (**b**) Keyence VHX-6000 digital microscope for bright-field observations. (**c**) A live butterfly sample set at the Keyence system. (**d**) Nikon A1^+^ inverted confocal laser microscope for fluorescent observations.

The pupal wing tissues are found on the surface of the pupal body in lepidopteran insects (Figure 2a). Immediately after pupation (within 30 min postpupation), the pupal forewing was lifted under a conventional dissection stereomicroscope (Figure 2b–d, Figure A2, Figure A3a; Video S1); when the pupa was still soft, the marginal area of the forewing was peeled up toward the wing base. A piece of cover glass (24 mm × 40 mm; 0.12–0.17 mm in thickness) (MUTO PURE CHEMICALS, Tokyo, Japan) was placed onto the exposed wings. The wings naturally stuck to the glass. For observations using an upright microscope, because the pupal body was under the glass, the glass was physically supported so that its weight did not harm the pupa. An operated pupa could develop natural color patterns in its wings (Figure 2e, Figure A2f) and occasionally eclose successfully (Figure 2e). It was possible to lift both the forewing and hindwing simultaneously so that the ventral hindwing could be observed (Figure 2d, Figure A2g). In this case, the exposed body surface was covered with a piece of plastic film to prevent the operated pupa from drying. Operations were performed at ambient temperature (26–28 °C) and at ambient humidity (40–80%). Imaging were performed at similar temperature but with more restricted humidity level (40–60%).

For dye loading, a droplet of 5–6 μL of solution containing fluorescent dyes was sandwiched between the forewing and hindwing and was left untouched for 30–60 min (Figure A2h). The wings were then washed 3 times with insect Ringer’s solution (10.93 g NaCl, 1.57 g KCl, 0.83 g CaCl_2_·2H_2_O and 0.83 g MgCl_2_·6H_2_O per liter) and the ventral forewing surface and dorsal hindwing surface were then placed on the piece of cover glass as explained above. Observations were readily made after the dye loading.

The observation of the ventral forewing is preferred to observe color pattern development because it has distinct black spots. In many species, observations of the ventral forewing have been difficult because the kink of the wing base in the forewing-lift configuration often causes extensive damage on the forewing. In the pale grass blue butterfly, we discovered that the operated forewing could survive well to the adult stage, making it possible to observe the entire ventral forewing (except the very basal area) during the entire developmental time course from immediately after pupation to eclosion. The dorsal hindwing has a curvature, which made it difficult to observe the whole wing areas at once without focus adjustment. By contrast, the ventral forewing could entirely be in contact with a glass plate, making it possible to observe the whole wing area at once with a fixed focal distance. The hindwing observations were also made as necessary. The survival rate of operated pupae to the adult stage with and without eclosion was 92.9% (*n* = 85) under our operations. 

### 2.4. Fluorescent Dyes

We employed the following four fluorescent dyes that were permeable to wing epithelial cells for confocal microscopic observations: Hoechst 33342 (Dojindo Molecular Technologies, Kumamoto, Japan) for nuclear staining; MitoRed (Dojindo Molecular Technologies) for mitochondrial staining; CFSE (5- or 6-(*N*-Succinimidyloxycarbonyl)fluorescein 3’,6’-diacetate) (Dojindo Molecular Technologies) for cytoplasmic staining, although it also stained nuclei in our system; BODIPY FL C_5_-ceramide complexed to BSA (Thermo Fisher Scientific, Tokyo, Japan) for staining of the Golgi apparatus, although it also stained the plasma membrane and other membranous structures in our system. We used the following final concentrations of fluorescent dyes: Hoechst 33342 (200 μM), MitoRed (1 μM or 10 μM), CFSE (100 μM) and BODIPY FL C_5_-ceramide (50 μM or 100 μM). The three dyes were often mixed together to make these final concentrations for simultaneous observations. The fluorescent dyes were first diluted with DMSO (dimethyl sulfoxide) and adjusted to the final concentration with insect Ringer’s solution. The excitation wavelengths of laser beams for these dyes were as follows: Hoechst 33342 (405 nm), CFSE and BODIPY FL C_5_-ceramide (488 nm) and MitoRed (561 nm). The emission filter wavelengths used to obtain confocal images were as follows: Hoechst 33342 (450/25 nm), CFSE and BODIPY FL C_5_-ceramide (520/25 nm) and MitoRed (595/25 nm). 

### 2.5. Microscopes, Image Acquisition, Editing and Analyses

We used a Keyence high-resolution bright-field digital microscope VHX-1000 or VHX-6000 (Osaka, Japan) for whole-wing monitoring (Figure A3b,c). Time-lapse images were taken in 1-min intervals (except for the compartmental images, in which case images were taken every 2 min) and compiled to 30-fps movies using open resource software FFmpeg 4.0 (The FFmpeg project; www.ffmpeg.org). The original image numbers (IN) are shown in figures; these image numbers (e.g., IN2000) directly correspond to minutes (e.g., 2000 min) after starting image acquisition but not postpupation time (e.g., 2000 min plus 9 h before starting). Epithelial areas were quantified using ImageJ (version 1.51i, Java 1.8.0_172) [35]. Using the compartmental-level images, 5 scale buds were randomly chosen at each time point and their sizes were measured using ImageJ [35]. To make figures, images were arranged using Adobe Photoshop Elements 11 (Adobe Systems, San Jose, CA, USA), but they were not modified except for the scale evagination images, for which contrasts were enhanced in grayscale images to identify individual cellular evagination.

For confocal laser microscopy, we used a Nikon A1^+^ confocal microscope system (Tokyo, Japan) equipped with a high-resolution Galvano scanner, built in a Nikon inverted epifluorescence microscope Eclipse Ti-U and operated by Nikon NIS Elements Imaging software (Tokyo, Japan) (Figure A3d). Using this software, epithelial 3D images were constructed from optical cross-sections of 0.4-μm steps from the apical surface along the *Z*-axis. The whole wing area just fits the low-power visual field of the microscope with a 4× objective lens. For high-power observations, a 100× oil-immersion objective lens was used. Acquired confocal images were painted with pseudocolors that match fluorescent colors of indicators used except for Hoechst 33342, in which case dark blue was replaced with cyan by adjusting the blue channel level in RGB mode using Adobe Photoshop.

## 3. Results

### 3.1. Whole Wing Dynamics and Peripheral Adjustment

**Figure 3:**
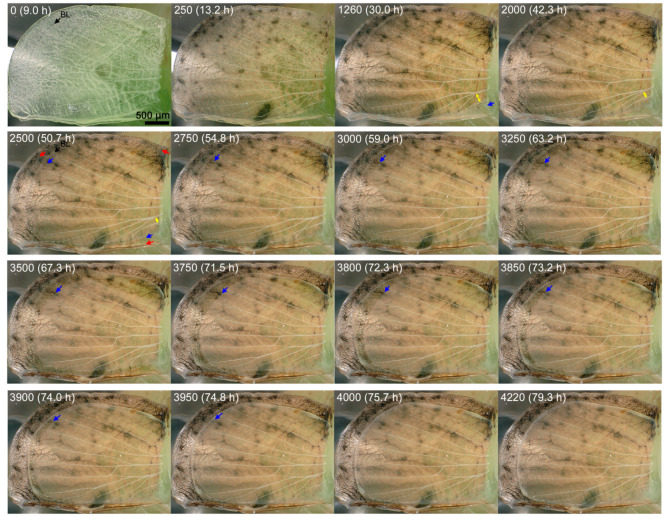
Dynamics of the pupal forewing. The forewing tissue was observed from the ventral side, distal to the left and anterior to the bottom. Image number and postpupation time are shown in each panel. Images were taken every 1 min and the first image taken was set at IN0. The image acquisition started at 9 h after pupation. The bordering lacuna (BL) is indicated in IN0. The edge of the ventral epithelium (blue arrows) and that of the dorsal epithelium (red arrows) are indicated. As an example, the distance between two tracheae is indicated by double-headed yellow arrows in IN1260–IN2500. Additionally, see Figure 4 and Figure 5 and Video S2.

**Figure 4:**
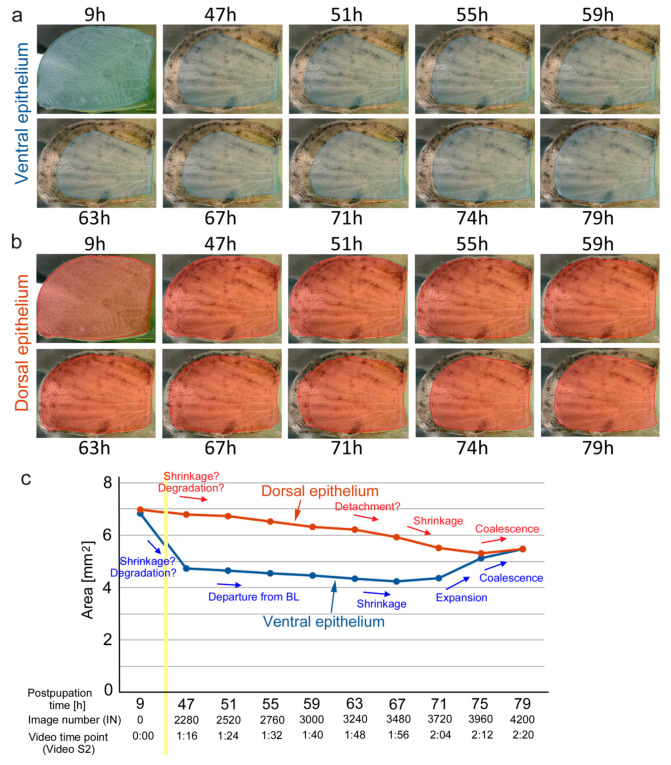
Time-dependent changes in the epithelial area in the developing pupal forewing. (**a**) Ventral epithelium. The areas measured are shown in blue. (**b**) Dorsal epithelium. The areas measured are shown in reddish brown. (**c**) Quantitative area changes. The events observed in the outer margin of the wings are indicated in arrows and with brief descriptions. Peripheral changes at the costal margin and hind (inner) margin are earlier than those at the outer margin. The vertical yellow bar indicates a time gap. Image numbers and time points in Video S2 are also indicated.

**Figure 5:**
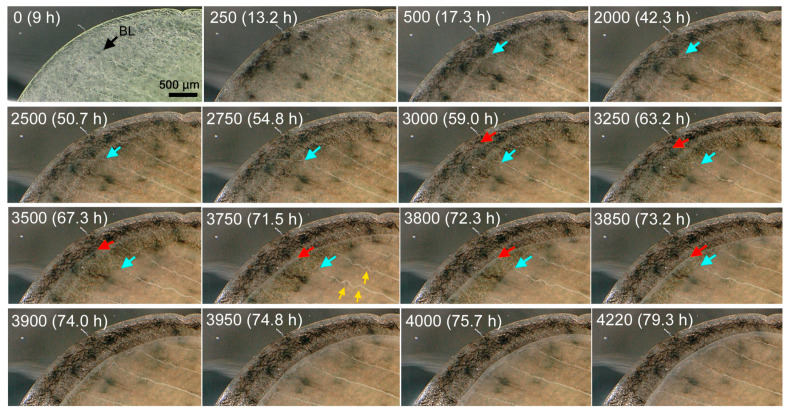
Dynamics of a distal area of the pupal forewing (enlarged images from Figure 4). BL in the panel IN0 indicates the bordering lacuna. The edges of the ventral forewing (blue arrows) and those of the dorsal forewing (red arrows) are indicated. The loosened tracheae are indicated by yellow arrows in IN3750.

**Figure 6:**
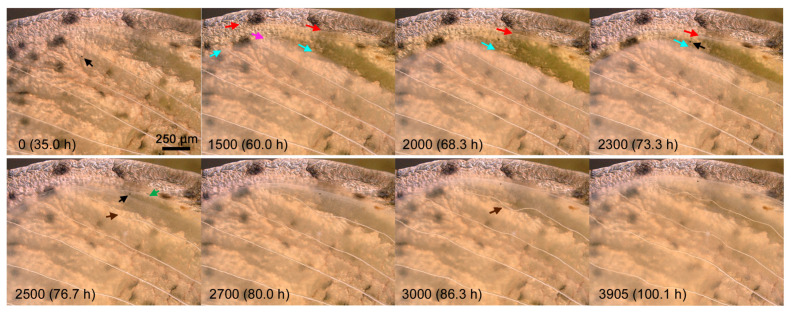
Dynamics of the pupal forewing along the hind (inner) margin (right) and outer margin (left). This is a different individual from that in Figure 3, Figure 4 and Figure 5. The image number and postpupation time are indicated in each panel. The edges of the ventral forewing (blue arrows), edges of the dorsal forewing (red arrows), bent trachea (a pink arrow in IN1500), loosened tracheae (brown arrows in IN2500 and IN3000) and a representative hemocyte at the wing margin (a green arrow in IN2500) are indicated. Among hemocytes, a large black one, often seen also in the other two individuals, is indicated by black arrows in IN2300 and IN2500. See also Video S3.

We first examined the whole forewing dynamics at high resolution images using a bright-field digital microscope (*n* = 3) (Figure 3, Figure 4, Figure 5 and Figure 6; Videos S2 and S3). In the first individual, which was reared at ambient room temperature throughout its life stages (Figure 3), the pupal cuticle was colored soon after pupation, but the wing tissue remained transparent for a while. The major venation system was already established immediately after pupation; the bordering lacuna was identifiable at 9-h postpupation (Figure 3, IN0). Some tracheae crossed the bordering lacuna and extended to the peripheral portion, whereas other tracheae were bent along the wing edge (IN0–IN250). The peripheral portion was rich in hemocytes and it might be degraded slowly before this time point, although we could not recognize the degradation process directly. Otherwise, there was no major change observed up until at approximately 30-h postpupation (IN1260) in the basal portion of the costal margin; the ventral epithelium began to move inward perpendicular to the wing margin. This was likely followed by similar movement of the basal portion of the dorsal epithelium approximately 0.5 h later. The movement pushed the existing tracheae in the margin (IN2000). From this portion of the wing, the wing tissue became gradually semitransparent from approximately 47–50 h (IN2500), making it clear for us to identify the edge of the ventral epithelium and less clear than the dorsal epithelium. At this point, it was clear that these two epithelia had different sizes and behaved differently. After this time point (IN2500), the basal wing edge of the dorsal epithelium in the hind (inner) margin also became semitransparent and became clear for our recognition. Simultaneously, the ventral epithelium in the outer margin slowly departed from the bordering lacuna toward the wing base at approximately 47–50 h (IN2500). Interestingly, it was clear at this point that the previous location of the bordering lacuna did not correspond to either edge of the dorsal and ventral epithelia. This inward movement of the ventral wing edge continued until approximately 67 h (IN2500–IN3250). Similar inward movement may be observed in all three margins and it became clear in the hind (inner) margin by 51 h (IN2500), but the hind (inner) margin appeared to show further progress: the dorsal epithelium began to shrink from approximately 42 h (IN2000) or even earlier. 

The outer margin of the dorsal epithelium also shrank from approximately 52 h first slowly and then relatively rapidly. The relatively slow change at the outer margin of the ventral epithelium was followed by relatively fast shrinkage of the ventral epithelium from approximately 59–67 h (IN3000–IN3500). While the dorsal epithelium was still shrinking, the ventral epithelium then expanded at approximately 72 h (IN3800). Epithelia coalesced and matched in size by 76 h (IN4000). The wing edge was clearly identified as whitish delineation. The coalescent epithelia then expanded slowly. While the hind (inner) margin of the dorsal epithelium was shrinking, numerous free-moving hemocytes were “trapped” at the edge of the moving epithelium, where they were crowded. The existing tracheae were pushed not only from the costal margin but also hind (inner) and outer margins, which changed the relative positions of tracheae and caused loosening (while the epithelia were shrinking) and stretching (while the epithelia were expanding) of tracheae. The whole wing areas were quantified at ten time points (Figure 4), although the wing edge was difficult to identify especially at earlier images. This quantification clearly showed that two epithelia had different area values and that they behaved differently before coalescence. 

The shrinkage and expansion of the epithelia were notable in enlarged snapshots of the posterior outer margin near the tornus in the same individual in reference to wing edges and tracheae (Figure 5). When the ventral forewing departed first from the bordering lacuna relatively slowly (IN2500), there was no clear change in tracheae and hemocytes. However, after the ventral forewing entered the relatively fast mode (IN3000), the entire tracheae were loosened considerably (IN3000–IN3750), but then they were stretched back (IN3850–IN4220). The movement of the dorsal epithelium might also accelerate the tracheal loosening and stretching. 

In the second individual, which was reared at ambient room temperature throughout its life stages, the same dynamics was observed (Figure 6; Video S3); focusing on the hind (inner) margin in this individual, we observed shrinkage of the ventral epithelium, followed by the dorsal epithelium. The shrinkage was accompanied by tracheal loosening (later stretching and bending at the margin) and hemocyte collection and crowding at the margin. Epithelial coalescence then occurred. Moreover, marginal scale elongation upon coalescence was clearly observed in this individual. Importantly, the very end of the coalescent wing margin did not seem to have scales. Movement of a large black hemocyte, probably a melanocyte, was also recorded clearly.

We also confirmed the whole wing dynamics in the third individual, which was reared at low temperature during the larval and prepupal stages. All dynamic characteristics were essentially the same as those of the previous two individuals; however, the entire process took approximately twice as long, although the image recording was performed at ambient temperatures as in other individuals: for example, this individual that experienced low temperature took 119.5 h for coalescence of the two epithelia, whereas the previous individuals took only 76 h. The coalesced epithelia then expanded, but they began shrinking at 136 h. The epithelia again expanded at 146 h to the end of this time-lapse imaging (168-h postpupation). 

### 3.2. Compartmental Dynamics and Scale-Bud Evagination

**Figure 7:**
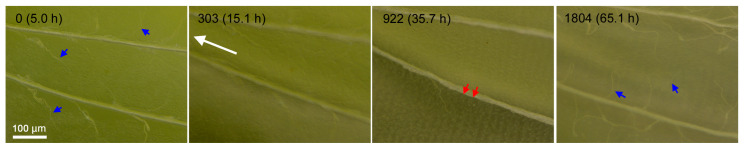
The M_3_ compartment dynamics in the dorsal hindwing. The postpupation time is indicated together with panel numbers. Images were taken every 2 min. Moving branching tracheae and tracheoles (blue arrows) and aligned scale arrays (red arrows) are indicated. The direction of tracheal movement to the base is indicated by a long white arrow. See also Video S4.

The wing dynamics should be understood not only at the whole-wing level shown above but also at the compartmental and cellular levels. We here examined the compartment-level dynamics at high-resolution images in the dorsal hindwing of an individual, which was reared at ambient room temperature using a bright-field digital microscope. We observed moving branching tracheae and tracheoles and moving hemocytes (Figure 7; Video S4). Alignment of epithelial cells was observed as early as 18-h postpupation and elongating scale buds at 36-h postpupation. The tracheae moved toward the base, but whether this tracheal movement is coordinated with the low-frequency contractions that have been observed in this time-lapse video (Video S4) and in the previous studies [16,20] or with the shrinkage for the peripheral adjustment that was observed in the forewing in the present study (Videos S2 and S3) is not understood. 

Because the entire hindwing moved vigorously due to the slow low-frequency contractions, the optical focus was difficult to maintain throughout the developmental stages. However, we could observe the contraction time course as follows. During the entire imaging period (5–171-h postpupation), the contractions were observed in the period between 14-h to 71-h postpupation. The number of contractions per hour changed over time; 5 times/h (14–15-h postpupation), 3–4 times/h (15–19 h), 1–2 times/h (19–65 h) and 2 times/h (65–71 h). Thus, the contraction frequency gradually faded from the earliest maximum. The earliest contractions at 14-h postpupation showed a relatively small amplitude and the amplitude increased as the contraction frequency became lower until 65 h. After this time point, the contraction amplitude decreased again as its frequency became lower. 

**Figure 8:**
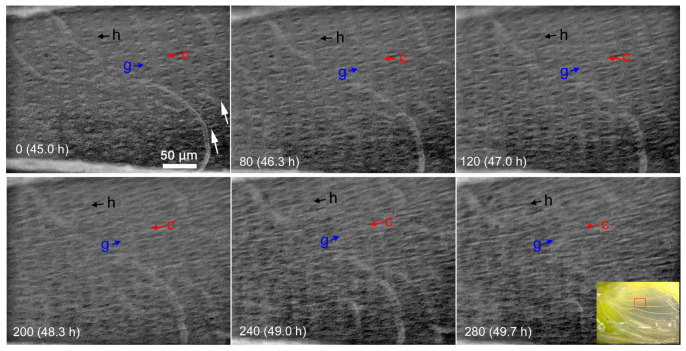
Scale and hair evagination in the CuA_1_ compartment of the dorsal hindwing. The image recording was started at 45 h after pupation. Images were obtained every 1 min; image numbers in each panel indicate minutes after the image recording was started. Colored arrows indicate a possible cover scale (red), ground scale (blue) and hair (black), respectively. White arrows in IN0 indicate arrays of aligned scale cells. The inset shows the location of the observation on the dorsal hindwing. See also Video S5.

**Figure 9:**
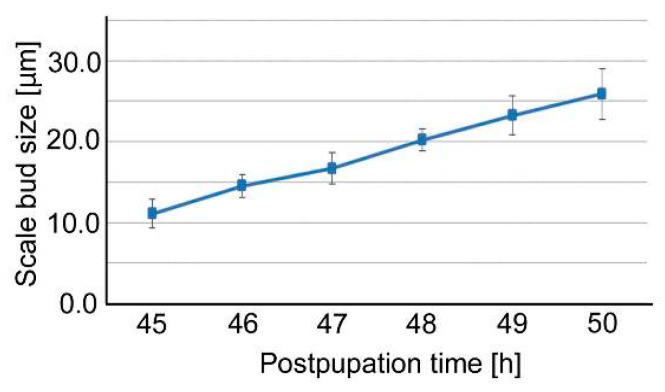
Size changes of the evaginating cover-scale buds. Each point and error bar indicate the mean ± standard deviation.

In more magnified compartmental images, we successfully recorded protruding cellular processes (i.e., scale buds) that contained elongating scales in a different individual (Figure 8; Video S5). Scale buds were present already in the hindwing at 45-h postpupation. The prospective cover scale and ground scale alternated in an array. Many prospective hairs were also noted. The size of the cover-scale buds changed over time; 11.1 μm (45-h postpupation), 14.5 μm (46 h), 16.7 μm (47 h), 20.2 μm (48 h), 23.3 μm (49 h) and 25.9 μm (50 h) (Figure 9). Thus, the velocity of evagination of the cover-scale buds varied from 2.2 μm/h to 3.5 μm/h in this 5-h period, but the overall velocity was 3.0 μm/h from 45-h to 50-h postpupation. We also obtained similar images of scale-bud evagination in the discal area of the ventral hindwing from a different individual (not shown).

### 3.3. Coloration Sequence

**Figure 10:**
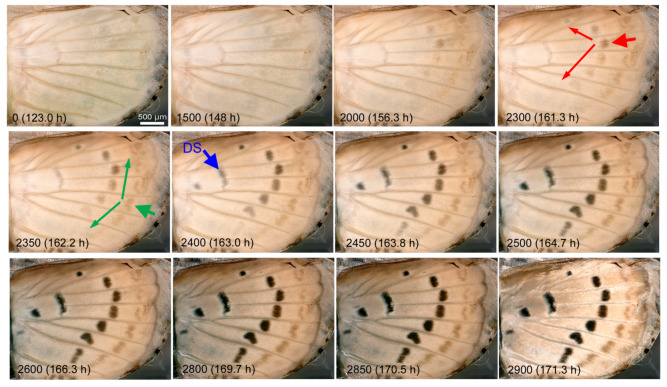
Coloration sequence of the pupal forewing. Images were recorded every 1 min; the image number and postpupation time are shown in each panel. The earliest emerging spot is clearly seen in IN2300 (indicated by a thick red arrow), although it can be seen as early as IN0. This is one of the spots in the third spot array. Gradients of the third spot array are indicated by thin red arrows in the same panel. In the second spot array, a spot in a different compartment emerges first that is indicated by a thick green arrow in IN2350. In the same panel, gradients of the second spot array are indicated by thin green arrows. The discal spot (DS), indicated by a blue arrow in IN2400, emerges later than other spots. See also Video S6.

We observed whole-wing changes at the late stages of the pupal wing development in an individual. No clear physical movement of the forewing was observed at this stage, but coloration change was notable. After the epithelial coalescence, the tissue further became semitransparent and then whitish (Figure 10; Video S6), indicating that scales were elongated by that time. Among the color pattern elements, the third spot array emerged first. Among the spots of the third spot array, the M_1_ spot emerged first, forming a gradient of emergence toward the anterior and posterior spots of the third array. Although the discal spot emerged later, this earliest M_1_ spot was located in the M_1_ compartment directly connected with the discal spot without interruption with wing veins, suggesting that the discal spot influences the third spot array. In the second spot array, the earliest spot was located in the M_3_ compartment, suggesting that the second and third spot arrays belong to different systems. 

### 3.4. Cellular Clusters and Dents of the Epithelium

**Figure 11:**
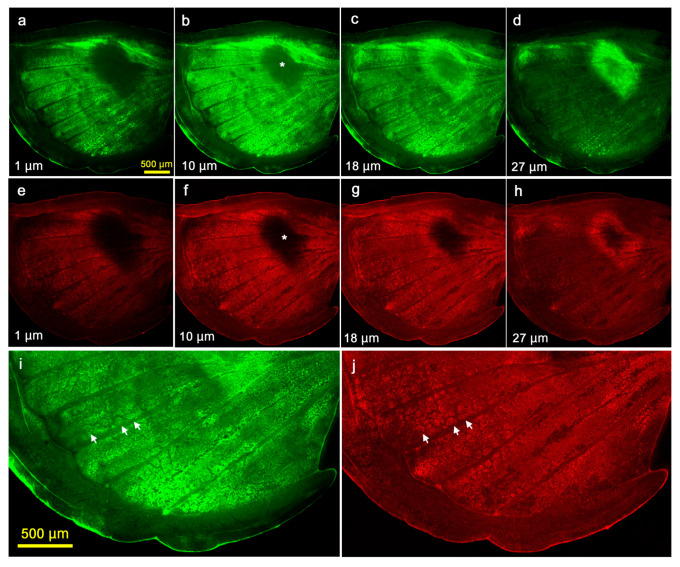
Serial optical sections of the dent at the prospective discal spot (the discal dent) and cellular clusters observed at the tissue level. The uppermost section near the apical epithelial surface is set at 0 μm. The depth from this section is indicated in each panel. Asterisks indicate the center of the discal dent. (**a**–**d**) CFSE staining for the cytoplasm. (**e**–**h**) MitoRed staining for mitochondria. (**i**) High magnification of c. Three representative clusters are indicated by arrows. (**j**) High magnification of g. Three representative clusters are indicated by arrows.

**Figure 12:**
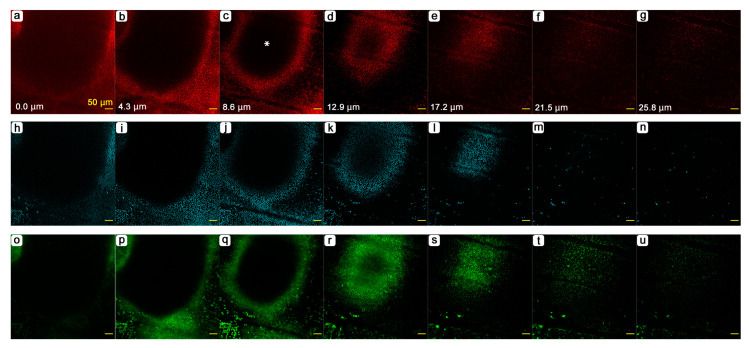
Serial optical sections of the dent at the prospective discal spot (the discal dent). This is a different individual from that in Figure 11. The uppermost section near the apical epithelial surface is set at 0 μm. The depth from this section is indicated in each panel in a-g; the three panels in the same column have the same depth. (**a**–**g**) MitoRed staining for mitochondria. An asterisk was placed at the center of the dent in c. (**h**–**n**) Hoechst 33342 staining for nuclei. (**o**–**u**) BODIPY FL C_5_-ceramide staining for the Golgi apparatus and membranous structures.

Hereafter, we examined the pupal forewing stained with fluorescent dyes using confocal laser microscopy. At the whole-wing level, we noted that there were some places where the ventral forewing epithelium was dented. The most conspicuous dent clearly corresponded to the prospective discal spot (Figure 11). The depth of an individual shown almost reached 30 μm using fluorescent dyes for the cytoplasm (Figure 11a–d) and mitochondria (Figure 11e–h), although the precise structure of the discal dent appeared to vary at least in two individuals examined in this study (see Figure 12). Additionally, staining with fluorescent dyes throughout the forewing made it possible to observe cellular distribution patterns throughout a wing. We discovered that epithelial cells were clustered at deeper levels throughout the wing although the wing surface was constructed by tightly packed epithelial cells (Figure 11i,j). Each cluster was separated by the hemolymph space, forming a mesh-like pattern. The discal dent was confirmed in another individual, where mitochondria, nuclei and Golgi and membranous structures were stained (Figure 12). The depth was approximately 20 μm in this case. 

**Figure 13:**
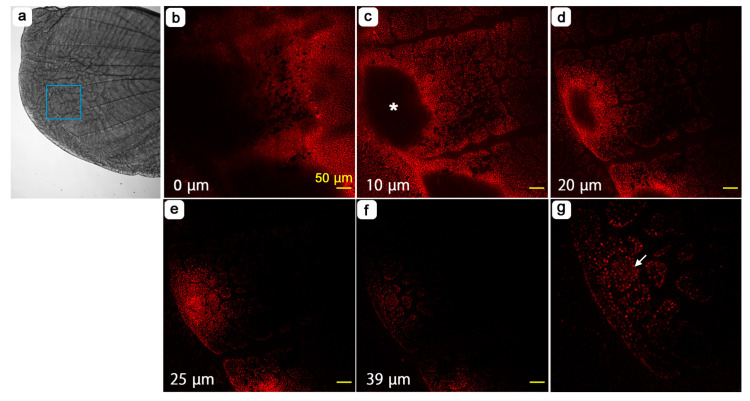
Dent at the wing margin identified by mitochondrial staining. (**a**) Bright-field image of the forewing. The region of interest in the following b-f panels corresponds to the blue square region. (**b**–**f**) Serial optical sections. The uppermost section near the apical epithelial surface is set at 0 μm. The depth from this section is indicated in each panel. An asterisk indicates the center of the marginal dent. (**g**) High magnification of the central area of the marginal dent shown in f. A cluster of nadir cells at the center of the dent structure is indicated by an arrow.

The marginal dents were also found at the center of a wing compartment along the wing margin (Figure 13). The marginal dent was almost as deep as 40 μm. At the bottom of the dent, a single cluster was located (Figure 12f,g), which was not clear in the discal dent. This cluster was thought to be composed of nadir cells [19].

### 3.5. Cellular Morphology and Distributions of Organelles

**Figure 14:**
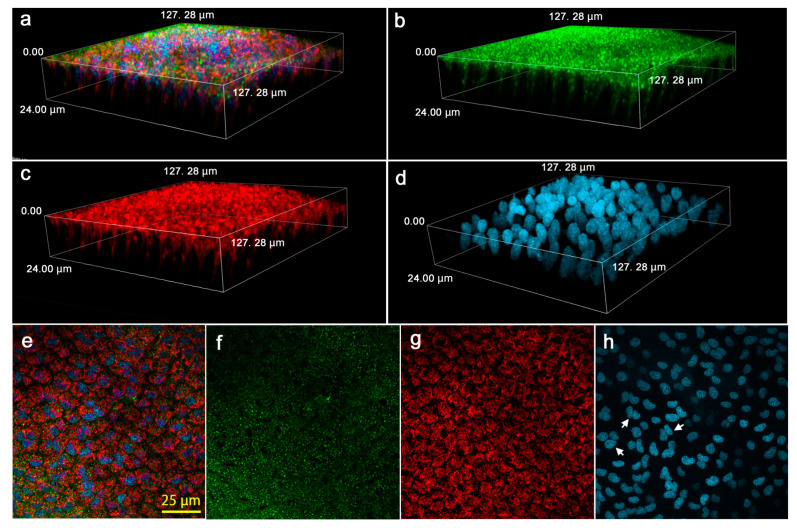
Cellular structures and organelles near the discal spot in the ventral forewing. (**a**) 3D image reconstitution of the ventral epithelium stained with three fluorescent dyes; Golgi apparatus and membrane (green), mitochondria (red) and nuclei (cyan). (**b**) 3D image of staining for the Golgi apparatus and membranous structure. (**c**) 3D image of staining for mitochondria. (**d**) 3D image of staining for nuclei. (**e**) 2D image of the ventral epithelium stained with the three fluorescent dyes. (**f**) 2D image of staining for the Golgi apparatus and membranous structure. In addition to the Golgi apparatus (numerous green dots), the plasma membranes are stained, indicating that epithelial cells are tightly packed. (**g**) 2D image of staining for mitochondria. (**h**) 2D image of staining for nuclei. Doublets and triplets are frequently observed (arrows).

Epithelial cells were visualized using three fluorescent dyes for Golgi and membranous structures, nuclei and mitochondria (Figure 14). Both the Golgi apparatus (Figure 14a,b) and mitochondria (Figure 14a,c) were distributed mostly near the apical surface, but they were also distributed at least down to 24 μm deep from the surface. At the surface, the Golgi, mitochondria and nuclei were observed (Figure 14e–h). The plasma membranes were stained (Figure 14f), showing tight packaging of cells at the apical surface in contrast to loose packaging at deeper levels. Many nuclei were doublets or triplets (Figure 14h), suggesting active nuclear division in the epithelial cells.

## 4. Discussion

In the present study, we analyzed live pupal wings of the pale grass blue butterfly *Z. maha* as a representative system for lycaenid butterfly wing development. As expected, many findings in the present study were similar to those previously found in the blue pansy butterfly *J. orithya*, one of the model nymphalid butterflies [16,17] (and in other butterflies [20] to a lesser extent), including peripheral adjustment, scale evagination and epithelial dents. These results suggest high levels of generality in wing development across families of Lepidoptera. The peripheral adjustment has been reported in many butterflies, especially in *Junonia almana*, in the previous study [20] but at low spatial and time resolutions. Here, we obtained whole-wing images of the entire peripheral adjustment process for the first time at high spatial and time resolutions.

The lycaenid system has some important advantages over the nymphalid system. First, the lycaenid system has a much smaller wing area than the nymphalid system. Thus, the entire wing area can be recorded comprehensively at once using a conventional low-magnification lens. Surprisingly, the “peripheral size adjustment” turned out to be the “whole-wing size adjustment” that involves the shrinkage and enlargement of both dorsal and ventral epithelia. Second, there appeared to be characteristics unique or exaggerated in a lycaenid butterfly; we discovered cellular clustering in this species that was less clear in nymphalid butterflies. Other methodological advantages of the pale grass blue butterfly such as the relative ease of mass rearing for experimental use [28] and abundance in the field throughout a year, at least in Okinawa, cannot be depreciated.

**Figure 15:**
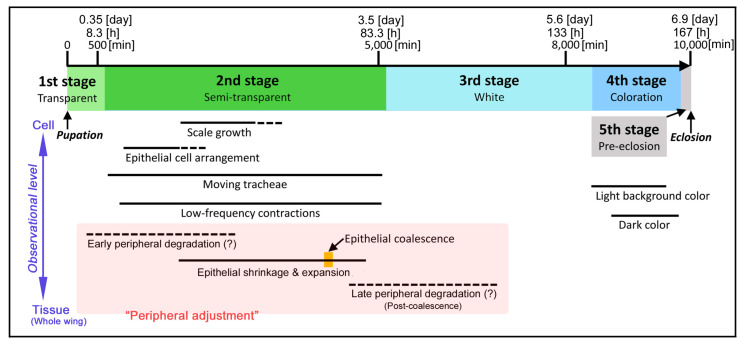
Time table of pupal wing development based on the present observations. Rough stages and time periods were indicated, assuming that the entire time span is approximately 10,000 minutes or 7 days. Several events of the epithelial movement and degradation are collectively called the peripheral adjustment, but it likely involves changes of the entire wings. Broken lines indicate that no clear observation was made.

Previously, we established that most butterfly species have the pupal period of approximately 10,000 min (7 d) and that the entire period of the pupal wing development can roughly be divided into four stages (plus the final fifth pre-eclosion stage of completed adult before emergence from the pupal case) based on the visual wing characteristics of twelve species [20]. The first stage is characterized by transparency and the second stage is semitransparency. Many important events such as color pattern determination, epithelial cell arrangement, peripheral adjustment and scale growth occur at the second stage. The third stage is the white-tissue stage and is relatively dormant. The fourth stage is the coloration stage. The pupal wings of the pale grass blue butterfly that were studied here roughly followed a similar sequence of events (Figure 15), but more detailed observations were made in the present study. Importantly, we observed developmental events at multiscale levels using the single system.

**Figure 16:**
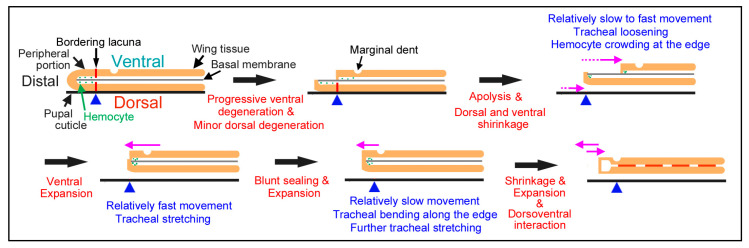
Schematic diagram for the peripheral adjustment of the pupal forewing. Possible changes are written in red and observations in the present study are in blue above or below illustrations. Directions of dynamic changes of epithelia are indicated by pink arrows. The locations of the bordering lacuna in the wing tissue are shown with red vertical bars and those in the pupal cuticle are with blue triangles, which serve as a guide spot, because it is safely assumed that the pupal cuticle does not move at all. Dorsoventral interaction is shown as horizontal orange bars between the ventral and dorsal epithelia. Dorsoventral interaction and blunt sealing are speculated (not observed here) based on the previous literature [37,38,42,43]. Cellular degradation is not confirmed in this study and, thus, is mostly speculative in this model. The coalescent edge of the two epithelia will be further degraded by programmed cell death (not shown in this diagram) [37,38,39,40].

One of the surprising discoveries in this study is that the peripheral adjustment involves complex movements of the dorsal and ventral epithelia. Based on the present results, we propose the following model of the epithelial movements (Figure 16). After an early static phase, the peripheral portion of the ventral (but not dorsal) epithelium may be degraded from the distal side slowly toward the bordering lacuna. The bordering lacuna has been considered to shape the wing edge that corresponds to adult wings [36,37,38,39,40,41]. The bordering lacuna may function as a blockade of the degradation wave. Being consistent with this, we observed the marginal dents located proximal to the bordering lacuna immediately after pupation, which would not be degraded. It is to be noted that, in this model, the peripheral portion of the dorsal epithelium does not experience extensive cellular degeneration. After this possible degradation, the edge of the ventral epithelium begins to depart from the bordering lacuna slowly toward the wing base. This was initially a relatively slow change for a short distance. 

After the initial departure of the distal edge of the ventral epithelium from the original bordering lacuna, the ventral epithelium soon began to shrink. This is unlikely due to degradation but likely due to physical shrinkage of the tissue because of the following three points; it is characterized by tracheal loosening, relatively fast and accompanied by hemocyte trapping and crowding at the wing edge. Following this shrinkage of the ventral epithelium, the dorsal epithelium now begins to reduce its size; the very edge of the peripheral portion moves toward the base. This shrinkage of the dorsal epithelium is also unlikely to involve degradation and would be executed after physical detachment of the dorsal epithelium from the pupal cuticle (i.e., apolysis). While the movement of the dorsal epithelium is still taking place, the ventral epithelium now expands and its distal edge meets with the distal edge of the dorsal epithelium. Interestingly, we observed that the loosened tracheae were stretched out again upon the expansion of the dorsal epithelium and that the tracheal distal portions beyond the original bordering lacuna were not degraded but simply bent at the right angle along the wing edge upon the coalescence of the dorsal and ventral epithelia. After the coalescence or even before this time point, the basal membrane that divides the two epithelial portions of hemolymph at the center may be degraded, as reported in previous histochemical literature [42,43] and the two epithelia would interact each other, probably to adjust the positions of radial lacunae and tracheae between the two epithelia. Because the very end of the coalescent wing margin does not have scales at this point, this narrow portion may also be degraded later. Indeed, it is likely that this degradation after the coalescence at the final stage of the peripheral adjustment corresponds to what has been reported in Figure 2 in Kodama et al. (1995) [38] and probably in other studies [37,39,40]. Unfortunately, we failed to focus on the edge during this period in time-lapse images in the present study.

The detailed analyses of the peripheral adjustment above questions conventional understanding of butterfly wing development. In *Drosophila*, the wing imaginal disc is a sac-like structure [9,10], meaning that the dorsal and ventral epithelia are continuous seamlessly at the margin. The butterfly wing disc is also a sac-like entity at the early stage [11]. However, in butterflies, it has been reported that the two epithelia are both blunt-ended in adult wings [37]. Furthermore, in butterfly pupal wings, it is believed that the peripheral area degenerates and the wing proper is excised, a process that is called a cookie-cutter mechanism [37,38,39,40]. Nonetheless, in Dohrman and Nijhout (1988) [37], the original paper that proposed this mechanism, the cross sections of the pupal wing margin of *Junonia coenia* at 48 h and 72 h after pupation were presented (Figure 2 in Ref [37]), where the dorsal and ventral epithelia are continuous. This is in contrast to the present study, but there is a possibility that the presented snapshots show just before and after the epithelial movements detected in the present study. The cross-sectional sequence presented in Kodama et al. [38] probably also missed the critical dynamic period, similar to Dohman and Nijhout (1988) [37]. Being consistent with the present study, in the snapshot of the 72-h cross section [37] and in cross sections in Kodama et al. [38], crowding hemocytes were found at the wing edge. Interestingly, in the 72-h or 3-d section [37,38], the wing edge epithelium is likely composed of small flat cells, which probably correspond to the coalescent edge that was observed as the narrow white margin without scales in the current study. Therefore, it appears that the early stage of the peripheral adjustment before coalescence has not been recognized by researchers until now. It also means that the fate of the peripheral portion distal to the bordering lacuna immediately after pupation has largely been unknown.

The epithelial movements appear to be autonomous. This contrasts with the wing tissue expansion during pupation; the wing tissue expands due to the pressure of hemolymph between the dorsal and ventral epithelia. Moreover, the epithelial movements detected in this study are not synchronized among the costal, hind (inner) and outer margins, indicating that epithelial portions are independent of one another. The epithelial sheet likely has physical tension created by tightly packed cells, as shown by fluorescent confocal images in this study and the movements of epithelia may be driven by cellular actin fibers. These speculations are consistent with the findings that wing veins were connected with actin fibers that may cause physical tension [41]. The surprisingly complex peripheral adjustment might have evolved only in a lineage of Lepidoptera for flexible wing shapes—for example, in response to environmental temperatures—and for independent coloration of the dorsal and ventral sides. 

The pupal wing tissue also shows slow low-frequency contractions, especially in the hindwings, as shown in the hindwing images in this study and in previous studies [16,20]. We noted that contractions change over time from relatively high frequency (5 times/h) and low amplitude to relatively low frequency (1–2 times/h) and high amplitude. The contractions are associated with an increase in the wing area in *J. orithya* [16]. More detailed analyses on the contractions are required to understand this movement. The contractions are clearly different from the epithelial movements for the peripheral adjustment, but their relationship is to be clarified in the future. 

The coloration sequence recorded in high spatiotemporal resolutions in *Z. maha* revealed that one of the black spots at the third spot array emerged first. This is a confirmation of the previous study [20], but it is somewhat unexpected from the previous discovery that the discal spot, which is considered the marking of the central organizer, is the earliest to emerge in some nymphalid butterflies [20], as expected from a possible function of the discal spot organizer. Moreover, it has already been suggested, based on a mutagenesis study, that the central symmetry system of *Z. maha* is composed of the third and fourth spot arrays and the discal spot and that the discal spot organizer organizes the location of these spots around it [24,25]. This organization of the central symmetry system is not necessarily reflected in the emerging order of black spots. Nonetheless, we discovered that the black spot located in the same wing compartment with the discal spot is the earliest spot to emerge, suggesting the direct influence from the discal spot organizer. From this spot toward the anterior and posterior sides, gradients of emerging spots were observed, suggesting that morphogenic signal gradients may exist. Equally interesting, the coloration sequence of the second spot array was different from that of the third spot array, suggesting that these two arrays belong to different color pattern systems.

Surprisingly, the color pattern determination occurs in the dynamic wing epithelia at the early stages after pupation [20]. Morphogenic signals should resist not only the hemolymph movement in the extracellular space and the slow low-frequency contractions but also the expansion and shrinkage of the epithelial tissue itself. Molecular morphogens such as Wnt cannot diffuse freely to make a stable gradient in such an unstable extracellular space. Nijhout (1991) [11] speculated that molecular morphogens may be transferred directly between neighboring cells via gap junctions or epidermal feet. We have speculated that molecular morphogens are likely transferred directly via cytonemes or a similar structure in butterflies [17,18,19,20]. In the present study, we confirmed that epithelial cells are tightly packed at the apical surface but loosely packed at deeper levels. We also discovered extensive cellular clusters that were distributed throughout a wing that may be a functional unit for molecular sharing, fate determination and differentiation. Clusters appear to exist in nymphalid butterflies, but clusters are much less clear in *J. orithya* [17]. More observations are necessary to delineate cellular and subcellular structures. 

The locations of organizers are “depicted” as spots and marks on the pupal wing cuticle in nymphalid butterflies [44,45,46]. The pupal wing cuticle of lycaenid butterflies may also show similar structures. Because the discal dent and the marginal dents were found in live tissues in the present study, their functions may be monitored in real time. In this sense, the present study set a foundation to examine, at cellular and tissue levels, the validity of the distortion hypothesis, which proposes that epithelial physical distortions function as morphogenic signals (i.e., nonmolecular morphogens) [47,48]. In a broader context, the induction model for the color pattern determination may also be examined [15,47,48,49,50,51,52,53].

Admittedly, there is still much room for technical improvement. Our time-lapse imaging for compartmental level was not very successful in that maintaining a fine focus on the surface of the wing was not attained. Either automated focus-tracking system or continuous human effort to adjust the focus throughout a recording period may be required for the entire pupal stage—i.e., 10,000 min (7 d) [20]. Additionally, the transparency of the wing at the early stage made it difficult to focus on the wing surface automatically. An additional problem of the current imaging system of butterfly pupal wings is that the fluorescent-dye loading and subsequent observations can be performed only once for a given individual and the fluorescent signals may be short term, probably because of bleaching, cellular degradation and dilution upon cell division. To trace cell fate and lineage, genetically encoded fluorescent probes such as green fluorescent protein (GFP) may be used. The GFP gene can be transferred and expressed in butterfly pupal wings using a baculovirus-mediated gene transfer method [54,55]. More fundamentally, the influence of the 13°C treatment on the delay of the pupal development must be characterized in detail. 

In the future, confocal studies will reveal details of cellular and subcellular structures. Functional imaging such as calcium imaging should also be performed, as well as more challenging imaging studies—for example, examining the distortion hypothesis for color pattern determination. Actin filaments may be illuminated during the shrinkage and expansion of the epithelial sheets, which may help to demonstrate physical tension on the epithelial sheet. Biophysically, super-resolution microscopy (SRM) may be employed to resolve organelle structures and how scale nanoarchitecture is formed in vivo in real time. 

## 5. Conclusions

This study demonstrated that the pupal wings of the pale grass blue butterfly *Z. maha* were suitable for live-image analyses of their development at multiscale levels. Our findings here in this small lycaenid butterfly confirmed the previous findings made by similar imaging analyses in the nymphalid butterfly *J. orithya* such as the peripheral adjustment, growing scales, tracheal development, hemocyte movement and cellular morphology. However, the entire dynamics of the peripheral adjustment was analyzed in detail, casting a question for conventional understanding of butterfly wing development made by static images. Extensive cellular clusters and epithelial dents, which may function during development, were discovered and their functional analyses may be expected. More improvements of the imaging system may further facilitate obtaining biologically critical images for developmental processes such as color pattern determination and scale formation in the future. 

## Supplementary Materials

The following videos are available online at https://www.mdpi.com/2313-433X/5/4/42/s1. Video S1: Surgical procedure, Video S2: Forewing dynamics (1), Video S3: Forewing dynamics (2), Video S4: Compartmental development, Video S5: Scale growth, Video S6: Coloration sequence.

## Appendix A

Rearing procedures used in this study. Rearing procedures are further described here (Figure A1) for reproducible rearing of this and other related species. The original method of Hiyama et al. (2010) [28] was slightly modified in the present study.

## Appendix B

Surgical procedures used in this study. Surgical procedures are further described here (Figure A2) for reproducible surgery of this and other related species. The forewing-lift method was also described in the previous papers [12,13,14,15,16,17,18,19,20], but they have mostly used nymphalid butterflies.

## Appendix C

Optical instruments used in this study. Optical instruments are further described here (Figure A3) for reproducible imaging results of this and other related species. We used Keyence and Nikon systems, but other similar commercially available systems will perform similarly.
